# Efficient Enrichment of Bacterial mRNA from Host-Bacteria Total RNA Samples

**DOI:** 10.1038/srep34850

**Published:** 2016-10-07

**Authors:** Nikhil Kumar, Mingqun Lin, Xuechu Zhao, Sandra Ott, Ivette Santana-Cruz, Sean Daugherty, Yasuko Rikihisa, Lisa Sadzewicz, Luke J. Tallon, Claire M. Fraser, Julie C. Dunning Hotopp

**Affiliations:** 1 Institute for Genome Sciences, University of Maryland School of Medicine, Baltimore, MD 21201, United States of America; 2 Department of Veterinary Biosciences, College of Veterinary Medicine, The Ohio State University, Columbus, OH 43210, United States of America; 3 Department of Medicine, University of Maryland School of Medicine, Baltimore, MD 21201, United States of America; 4 Department of Microbiology and Immunology, University of Maryland School of Medicine, Baltimore, MD 21201, United States of America.

## Abstract

Despite numerous advances in genomics and bioinformatics, technological hurdles remain to examine host-microbe transcriptomics. Sometimes the transcriptome of either or both can be ascertained merely by generating more sequencing reads. However, many cases exist where bacterial mRNA needs to be enriched further to enable cost-effective sequencing of the pathogen or endosymbiont. While a suitable method is commercially available for mammalian samples of this type, development of such methods has languished for invertebrate samples. Furthermore, a common method across multiple taxa would facilitate comparisons between bacteria in invertebrate vectors and their vertebrate hosts. Here, a method is described to concurrently remove polyadenylated transcripts, prokaryotic rRNA, and eukaryotic rRNA, including those with low amounts of starting material (e.g. 100 ng). In a *Wolbachia-Drosophila* system, this bacterial mRNA enrichment yielded a 3-fold increase in *Wolbachia* mRNA abundance and a concomitant 3.3-fold increase in the percentage of transcripts detected. More specifically, 70% of the genome could be recovered by transcriptome sequencing compared to 21% in the total RNA. Sequencing of similar bacterial mRNA-enriched samples generated from *Ehrlichia*-infected canine cells covers 93% of the *Ehrlichia* genome, suggesting ubiquitous transcription across the entire *Ehrlichia chaffeensis* genome. This technique can potentially be used to enrich bacterial mRNA in many studies of host-microbe interactions.

Many important biological interactions and diseases arise from a diverse variety of obligate intracellular bacteria. This may be best epitomized by the bacteria in the order Rickettsiales, which includes several Category ABC *Rickettsia* pathogens that cause Rocky Mountain spotted fever, typhus, and other spotted fevers^1^. This order also includes *Orientia tsutsugamushi* that causes scrub typhus^1^, *Ehrlichia chaffeensis* that causes Ehrlichiosis^2^, and *Anaplasma phagocytophilum* that causes Anaplasmosis^2^, as well as many endosymbionts of arthropods, including *Rickettsia* endosymbionts^3^,^4^ and the prolific *Wolbachia* endosymbionts^5^,^6^. The Rickettsiales order illustrates the connection between pathogenesis and endosymbiosis. For example, it has been proposed that *Ehrlichia chaffeensis* and *Anaplasma phagocytophilum* may be endosymbionts of ticks but pathogenic to humans^7^. Given the interest in, and the complexity of, the biological interactions in this clade, we sought to develop a method to enrich for bacterial transcripts prior to transcriptome sequencing in mixed RNA samples that we could use specifically on studies of Rickettsiales organisms and both their vertebrate and invertebrate hosts. In particular, we focused on the *Wolbachia* endosymbiont of *Drosophila ananassae w*Ana and its invertebrate fruit fly host, *Ehrlichia chaffeensis* Wakulla infected canine cell culture, and the *Wolbachia* endosymbiont of *Brugia malayi w*Bm and its invertebrate nematode host.

*Wolbachia* endosymbionts are intracellular endosymbionts of many arthropods and filarial nematodes^5^,^6^. *Wolbachia* strains can induce parthenogenesis, male killing, feminization, and cytoplasmic incompatibility in arthropods and can be mutualistic in some insects and filarial nematodes^5^,^6^. The phenotypes induced by *Wolbachia* strains have led to several studies on their use as biocontrol agents targeting arthropod vectors^8^,^9^ and reduction of viruses in those vectors^10^. Many *Wolbachia* hosts have also been found to have extensive lateral gene transfer from the bacteria to the host^11^,^12^, including the *Drosophila ananassae* line from Hawaii^13^,^14^ and the line from Malaysia^13^ that was used in this study.

*Ehrlichia chaffeensis*, the causative agent of Ehrlichiosis, is a bacteria found in the lone star tick, *Amblyomma americanum*^2^. Ehrlichiosis, which was designated a nationally notifiable disease in the US in 1998^2^, is characterized by flu-like symptoms with severity that ranges from asymptomatic seroconversion to death^2^. Ehrlichiosis is most frequently reported from the southeastern and south-central United States. Research on this bacteria is complicated by its complex life cycle. In addition to having obligate associations with humans in which it causes disease, *Ehrlichia* is found in tick vectors and in vertebrate reservoirs, such as white-tailed deer^2^.

Previously, we described a method that efficiently removes >95% of insect rRNA from total RNA samples obtained from a *Drosophila ananassae* Hawaii line^15^. This resulted in a 6.2-fold increase in mRNA abundance^15^. However, this merely depletes the rRNA, not abundant host mRNA in the sample. When microbial RNA is relatively abundant compared to the RNA of the eukaryotic hosts, the two can be efficiently sequenced simultaneously^16^. Yet, in many cases the bacterial RNA is far less abundant and needs to be further enriched. For example, the total RNA from *Wolbachia*-colonized *Drosophila ananassae* has only a very small amount of bacterial 23S rRNA that can be detected using an Agilent Bioanalyzer (Fig. 1). The bacterial mRNA in this total RNA sample is even less abundant than the rRNA molecules, presenting a challenge for cost-effective transcriptome sequencing-based experiments. Here, we examine if polyA enrichment techniques can be co-opted to deplete samples of polyadenylated eukaryotic mRNA, leaving the bacterial mRNA behind. When combined with the Ribo-Zero method presented previously^15^, the resulting RNA should be extensively enriched for bacterial mRNA that can then be cost-effectively sequenced to measure the bacterial transcriptome. To this end, we developed and tested a technique to generate rRNA-depleted, polyA-depleted RNA using the Ribo-Zero Gram-Negative Bacteria rRNA Removal Kit, the Ribo-Zero Human/Mouse/Rat rRNA Removal Kit, and the Invitrogen Dynabeads Oligo-dT mRNA isolation kit. In the latter case, the kit is intended to enrich for polyadenylated mRNA found in eukaryotes. Instead of discarding the supernatant and eluting from the polyA-enrichment substrate, the supernatant was retained. We show here that this supernatant can be used to enrich for bacterial mRNA in samples and will be referred to as bacterial mRNA-enriched samples. We used this technique to prepare samples successfully for analysis with the Agilent Bioanalzyer, qRT-PCR, and RNA-Seq. While this technique was tested on bacteria-animal systems, for which a viable commercial alternative has not been available, it can be used on any such sample where the rRNA kit can be used to deplete eukaryotic rRNAs like many invertebrates^15^, vertebrate animals, and fungi.

## Results

### Assessment of method on *D. ananassae* and *Wolbachia* endosymbiont RNA using microfluidics and qRT-PCR

**Figure 1:**
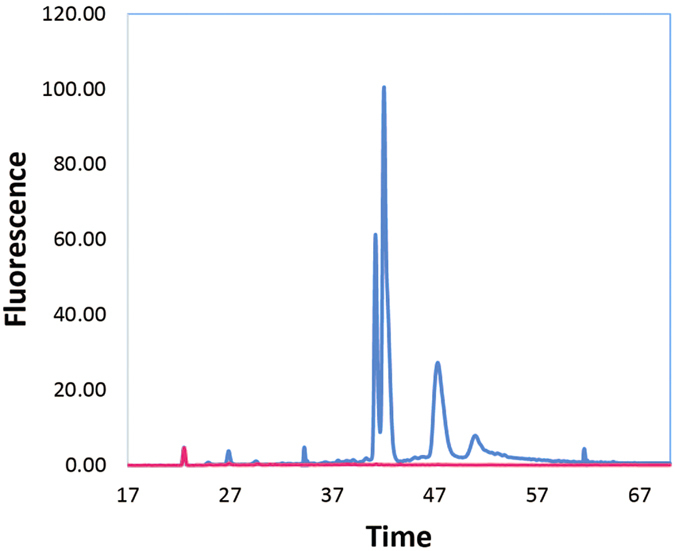
Bioanalyzer analysis of total RNA and bacterial mRNA-enriched samples from *Drosophila ananassae* colonized by its *Wolbachia* endosymbiont. The subtraction of *Drosophila* rRNA was assessed by running equivalent amounts of total RNA (**blue**) and Ribo-Zero reduced RNA (**pink**) on a Bioanalyzer. The software calculated the concentration of each sample by integrating the area under the rRNA peaks. Total RNA was 331 ng/μL and Ribo-Zero reduced RNA was 8 ng/μL, for an RNA loss of >97%, most of which is in the rRNA peaks for both the bacterial endosymbiont and invertebrate host.

We used both an Agilent Bioanalyzer and qRT-PCR to examine the relative abundance of the various transcripts in total RNA and in the bacterial mRNA-enriched RNA samples from *D. ananassae* from Malaysia (Table 1). In both cases, in order to enable direct comparisons, the template added reflects the same amount of starting material, as described in the methods. As expected, *Drosophila* rRNA removal by the Ribo-Zero Human/Mouse/Rat component was effective with a total loss of >97% of RNA, as assessed on the Agilent Bioanalyzer (Fig. 1). This percentage loss is consistent with our reported prior rRNA reductions in this system^15^. Because the insect 23S rRNA is cleaved and co-migrates with the insect 18S rRNA and the bacterial 16S rRNA, the *Wolbachia* 16S rRNA is obscured prior to rRNA depletion. However, following rRNA depletion, the *Wolbachia* 16S and 23S rRNA were not observed, nor were the insect 18S and 28S rRNA, indicating that they were all efficiently removed (Fig. 1).

To better quantify the extent of bacterial mRNA enrichment, qRT-PCR was conducted with primers designed to target bacterial rRNA, eukaryotic rRNA, bacterial mRNA, and eukaryotic mRNA, allowing for a comparison of all four types of molecules. The bacterial mRNA enrichment method efficiently increased the Ct values for *Wolbachia* 16S rRNA with the ΔCt ranging from −5.5 to −13.9, yielding a 97.7–99.9% depletion of the *Wolbachia* 16S rRNA (Table 1). Highly abundant *Drosophila* mRNAs were efficiently removed with the Dynabeads component of the bacterial mRNA enrichment method with the ΔCt ranging from −1.3 to −3.38, which corresponds to a reduction of 71% of Act5C transcripts and 87% of ribosomal protein L32 transcripts removed (Table 1). Yet, *Wolbachia* mRNA abundance was unchanged by the bacterial mRNA enrichment, as illustrated by the similar qRT-PCR Ct values in pre- and post-enrichment of the samples for *Wolbachia* mRNA, yielding a ΔCt near zero (ranging from −0.33 to 0.69) in the three replicates examined (Table 1). Each replicate is the result of a separate RNA extraction as well as enrichment.

### Assessment of method on *D. ananassae* and *Wolbachia* endosymbiont RNA using high throughput sequencing

Following the successful assessment of bacterial mRNA enrichment by qRT-PCR and microfluidics, three samples were prepared for Illumina MiSeq sequencing: total RNA, polyA-selected RNA, and the bacterial mRNA-enriched RNA. The MiSeq reads were mapped with BWA MEM^17^ to a database containing the reference *D. ananassae* genome and the *Wolbachia* endosymbiont genome from strain *w*Ri (NC_012416.1^18^) resulting in 193,612 (1.8%) *Wolbachia* reads and 8,889,348 (98.2%) *D. ananassae* reads mapping in the total RNA, 1,923 (0.02%) *Wolbachia* reads and 9,318,954 (99.98%) *D. ananassae* reads mapping in the polyA-enriched RNA, and 62,089 (1.0%) *Wolbachia* reads and 6,029,306 (99%) *D. ananassae* reads mapping in the bacterial mRNA-enriched samples, as reported by IDXSTATS in MPILEUP.

While the percentage of *Wolbachia* reads is not higher in the bacterial mRNA-enriched sample when compared to total RNA, it is important to remember that the bacterial rRNA has been depleted, indicating more of this percentage should arise from bacterial mRNA than in the other samples. Thus, while the reads from the total RNA sample and polyA-enriched RNA samples span 298 kbp (21%) and 66 kbp (4.7%) of the 1.4 Mbp *w*Ri reference *Wolbachia* genome, respectively, the reads spanned 977 kbp of the *w*Ri genome (70%) in the bacterial mRNA-enriched sample. Therefore, while the relative abundance of *Wolbachia* reads are the same in the polyA-enriched and bacterial mRNA-enriched samples, the latter is the only sample where bacterial transcripts can be detected across the majority of the genome.

### Comparisons of methods after normalization

**Figure 2:**
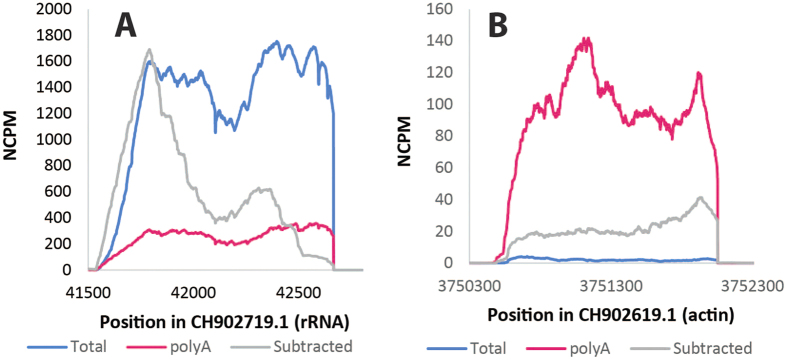
Depletion of *Drosophila* rRNA and actin transcripts. Coverage was compared for the *Drosophila* rRNA (panel A) and the actin gene (panel B) for the total (blue), polyA-selected (pink), and the bacterial mRNA-enriched (gray) RNA samples after normalizing for the number of reads sequenced, as calculated as NCPM, or normalized coverage per million reads sequenced. rRNA is highly abundant in the total RNA, but significantly reduced in the polyA-selected and the bacterial mRNA-enriched samples. In contrast, the actin transcript was enriched only in the polyA-enriched sample. Therefore the method of bacterial mRNA enrichment was effective at removing both eukaryotic mRNA and rRNA.

**Figure 3:**
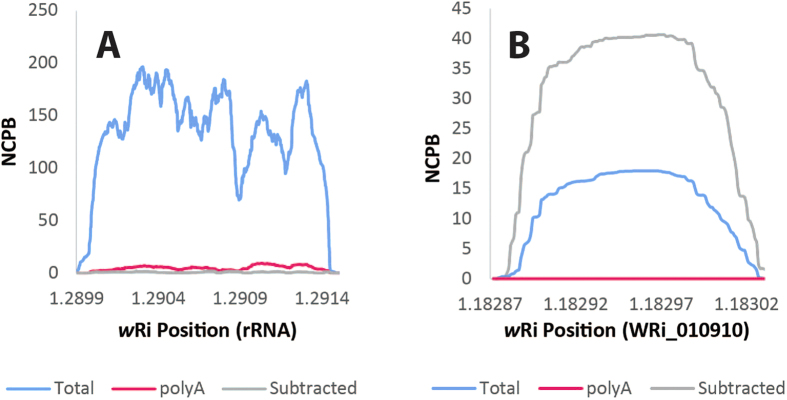
Depletion of *Wolbachia* rRNA and enrichment of *Wolbachia* mRNA. Coverage was compared for the *Wolbachia* rRNA (panel A) and the WRi_010910 gene (panel B) for the total (blue), polyA-selected (pink), and bacterial mRNA-enriched (gray) samples after normalizing for the number of reads sequenced, as calculated as NCPB, or normalized coverage per billion reads sequenced. rRNA was highly abundant in the total RNA, but significantly reduced in the polyA-selected and the bacterial mRNA-enriched samples. In contrast, the WRi_010910 transcript was enriched in the bacterial mRNA-enriched sample compared to the total RNA. Therefore the method was effective at enriching for bacterial mRNA.

To measure the fold change, the coverage needs to be normalized by the variable numbers of bases sequenced. Therefore, the coverage was normalized by dividing the sequencing coverage by the number of bases sequenced and multiplying by a million to generate the NCPM, or normalized counts per million reads sequenced. This value is analogous to RPKM or FPKM but on a genome scale as opposed to a gene scale. As expected, the *D. ananassae* rRNA was found in all samples, but relative to the total RNA was 5.2-fold decreased in abundance in the polyA-enriched RNA and 2.1-fold decreased in abundance in the bacterial mRNA-enriched sample (Fig. 2A), as assessed by integrating the area under the curve. For comparison, the actin transcript was 10-fold overrepresented in the polyA-enriched sample relative to total RNA and 33-fold underrepresented in the bacterial mRNA-enriched sample relative to total RNA, indicating that the polyA depletion was successful (Fig. 2B). Relative to the total RNA, *Wolbachia* rRNA was 64-fold underrepresented in the bacterial mRNA-enriched sample and 28-fold underrepresented in the polyA-enriched sample, demonstrating that the bacterial rRNA is reduced, but not eliminated, with both polyA enrichment and bacterial mRNA enrichment (Fig. 3A). Demonstrating the power of the method tested, the *Wolbachia* gene WRi_010910 was 2.7-fold more abundant in the bacterial mRNA-enriched sample than in total RNA and was completely undetected in the polyA-enriched sample. Transcripts could be detected from 70% of the genome in the bacterial mRNA-enriched sample, as opposed to just 21% of the genome in the total RNA sample. Therefore, this method simultaneously increases the number of transcripts detected and the number of reads/transcript.

### Assessment of method on *Canis lupus* and *Ehrlichia chaffeensis* RNA

**Figure 4:**
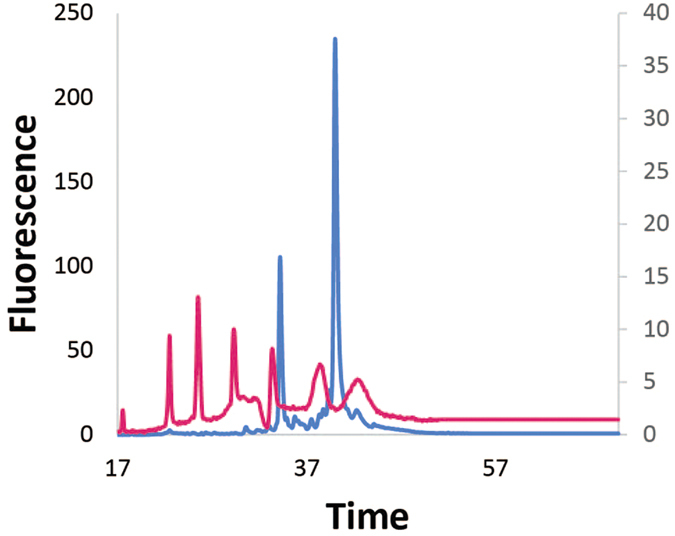
Bioanalyzer analysis of *Ehrlichia* and canine total RNA abundance. Using the Bioanalyzer, the *Ehrlichia-*canine total RNA (blue, left axis) was compared to the RNA 6000 ladder (pink, right axis), which contains 0.2, 0.5, 1.0, 2.0, 4.0, and 6.0 kbp fragments. Only canine rRNA was evident with no detectable bacterial rRNAs.

After determining the efficacy of the bacterial mRNA enrichment method using the model system *D. ananassae* on a partial Illumina MiSeq run yielding ~10 million reads, we sought to further test the method to enrich *Ehrlichia* mRNA in complex RNA mixtures sequenced with ~50 million reads on the Illumina HiSeq. Total RNA was isolated from DH82 *Canis lupus* cells infected with *Ehrlichia chaffeensis* Wakulla (Fig. 4).

Due to limited amounts of RNA, the samples were not examined by qRT-PCR or the Bioanalyzer following bacterial mRNA enrichment, and transcriptome sequencing was undertaken directly of the bacterial mRNA-enriched sample. Mapped reads spanned 1,098,477 of the 1,179,491 bp Wakulla genome, or 93.1% of the genome. Despite there being a lower abundance of bacterial RNA when compared to eukaryotic RNA in these samples (Fig. 4) relative to the *Wolbachia-Drosophila* samples (Fig. 1), the enrichment of *Ehrlichia* transcripts was better than that for *Wolbachia*, resulting in a higher percentage of reads mapping to the bacterial genome. This is likely due to better removal of vertebrate rRNA with the Ribo-Zero kit than insect rRNA.

**Figure 5:**
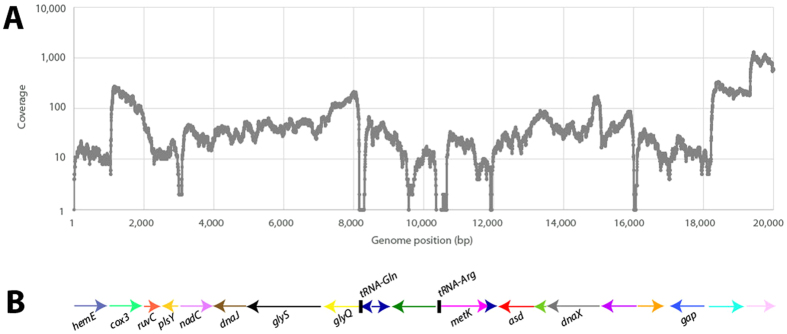
*Ehrlichia* transcriptome sequencing coverage across a genome segment. The bacterial mRNA-enriched transcriptome sequencing coverage was plotted for the first 20 kbp of the Wakulla genome (Panel A) and compared to the predicted genes for this region (Panel B). While 99% of the genome is transcribed, troughs are apparent for tRNAs, which are too small to be recovered with the RNA isolation method used here. The coverage peaks at the 5′-end of transcripts and decays over the length of the transcript, as has been observed for other bacterial transcriptomes. Troughs are seen at transcriptional start sites, but not always at the 3′-ends of transcripts. This suggests that either the 3′-end of the transcripts overlap the ends of other transcripts, or that this strain lacks discrete transcription termination sites.

The coverage varies in a manner consistent with the genes in the genome with coverage troughs at gene boundaries and tRNAs, the latter of which is lost during RNeasy purification (Fig. 5). Previously, it has been demonstrated by proteomics that 99% of proteins with known function and >80% of hypothetical proteins are expressed in *Ehrlichia chaffeensis* in infected human cells^19^. Simultaneous expression of all genes may be expected since the genome lacks transcriptional regulators relative to a free-living relative^7^, which in turn is consistent with a restricted intracellular life style. The ECH_0166 gene is the most abundantly transcribed gene, which is annotated as a hypothetical protein but has been shown to encode the immunoreactive protein TRP47, which is secreted during infection^20^,^21^.

### Assessment of low input method on RNA from *Brugia malayi* and its *Wolbachia* endosymbiont using qRT-PCR

While in many cases, it is possible to obtain 5 μg of RNA for use with the standard Ribo-Zero kits, for many biologically significant conditions this is a large amount of RNA to acquire. One such example is some of the specific life stages of filarial nematodes, like *Brugia malayi,* where material is limited. However, newer procedures for Ribo-Zero reductions allow the use of 100 ng of total RNA. Therefore, we sought to establish if the polyA removal would work efficiently on these low input samples. To this end, we tested a low input method for Ribo-Zero reduction and polyA subtraction using 100 ng of RNA from an adult filarial nematode and assessed the success using qRT-PCR.

As with the previous *Wolbachia-*host samples, to quantify the extent of bacterial mRNA enrichment, qRT-PCR was conducted with primers designed to target bacterial rRNA, eukaryotic rRNA, bacterial mRNA, and eukaryotic mRNA, allowing for a comparison of all four types of molecules (Table 1). Again, in order to enable direct comparisons, the template added to the qRT-PCR reactions reflects the same amount of starting material, as described in the methods. As expected, *Brugia* rRNA removal by the Ribo-Zero Human/Mouse/Rat component was effective, with a total loss of >99% of 18S rRNA, resulting in a ΔCt of −17 between the pre-subtraction RNA and the post-subtraction RNA (Table 1). The bacterial mRNA enrichment method efficiently decreased the Ct values for *Wolbachia* 16S rRNA with the ΔCt of −7.5, yielding a >99% depletion of the *Wolbachia* 16S rRNA (Table 1). Highly abundant *Brugia* mRNAs were efficiently removed with the Dynabeads component of the bacterial mRNA enrichment method, with the ΔCt of −2.4, yielding 76–84% reduction, for actin transcripts and a ΔCt of −3.5, yielding 88–94% reduction, for Bm1_03910 (Table 1). *Wolbachia* mRNA abundance for two genes tested (Wbm0276 and Wbm0350) yielded a ΔCt of −1.0 following bacterial mRNA enrichment, or a reduction of 47–55%, which we attribute to general loss of RNA with the low input method (Table 1). Therefore, we conclude that lower input starting materials can be efficiently examined with this method, albeit with a larger percentage loss of material.

## Discussion

### Cost savings

In sequencing from *Wolbachia* mRNA-enriched samples, we observe a 3-fold increase in reads aligning to the gene analogous to WRi_010910 and 3.3-fold more transcript sequence detected. This is a substantial improvement in the detection of bacterial mRNA. The added cost of conducting the Ribo-Zero and polyA depletion was $124 for the standard input samples and $65 for low input samples (Table 2) with the Dynabeads adding $46 to the cost of the sample. Of course, actual costs, as opposed to list prices, vary greatly by location and currency.

To obtain 10 million mapped bacterial reads, we estimate that the library and sequencing costs would total $12,325 for the *Wolbachia* samples and $1,165 for the *Ehrlichia* samples (Table 2). The 10-fold difference between the two samples relates to the efficiency of the Ribo-Zero depletion. The kit used was designed on the human, mouse, and rat rRNA. As described previously^15^, we are co-opting the use of this kit here for taxa on which it was not designed, specifically for removal of rRNA from canines and insects. Our results indicate that the kit performs better at removing canine rRNA than insect rRNA. Thus, this difference in depletion of rRNA leads to a difference in the sequencing costs to obtain 10 million mapped bacterial reads. Regardless, these techniques substantially decrease the cost of sequencing compared to total RNA, which we estimated to be around $600,000 to obtain 10 million mapped bacterial reads. The real costs to obtain equivalent data would actually be higher than $600 K since the vast majority of bacterial reads in total RNA will map to the bacterial rRNA. We did not include an estimate of the personnel time required to prepare the samples, but it is surely significantly less than the difference between the method described here to obtain bacterial mRNA enriched samples and the alternative, which is sequencing total RNA. As such, employing this technique should greatly enable transcriptome-based studies of bacteria-eukaryote interactions in the largely intractable bacteria in the order Rickettsiales, as well as other important taxa.

The magnitude of the removal of rRNA with Ribo-Zero subtraction kit has been a bit unpredictable as observed in the qRT-PCR results (Table 1). It is not clear where that variation arises, whether it is the quality of the total RNA, aspects of the kit, limitations of the qRT-PCR method, or user error. However, even with the lowest level of enrichment, the savings is significant given the alternative, which is sequencing total RNA.

Much of the cost savings likely comes from the rRNA depletion strategy, which was not included in the comparisons here. However, the relatively small cost of including the DynaBeads ($46) and the insubstantial amount of personnel hours added upon inclusion of this step means that it will be cost effective.

Recently, Agilent has introduced custom capture systems designed for capturing RNA, analogous to its systems for capturing DNA. For comparison, the cost of a custom capture of *Wolbachia* mRNA or *Ehlrichia* mRNA is estimated from the list price to be $563/sample. While this is substantially more than the cost of the bacterial mRNA enrichment method presented here, it may have benefits for samples where the rRNA depletion is less than ideal, like capturing bacterial mRNA from insect hosts. One study suggests a 1670-fold enrichment of RNA^22^, which would reduce the sequencing costs of the *Wolbachia* samples described here substantially to the levels of sequencing conducted on *Ehrlichia* and make the SureSelect the more cost effective option.

## Conclusions

Here, we describe a method to efficiently remove eukaryotic host mRNA from 100 ng and 5 μg of starting material through polyA depletion. Combined with Ribo-Zero reductions, which efficiently remove rRNA from bacteria and eukaryotes, more bacterial mRNA transcripts can be identified with higher coverage in a *Wolbachia-Drosophila* system. Sequencing of a bacterial mRNA-enriched sample isolated from a canine-*Ehrlichia* system results in 20% of the sequence reads arising from the bacterial transcriptome. In both cases, these methods enabled more cost-effective transcriptional profiling of host-bacteria samples than conventional methods. While both of the bacteria are from the order Rickettsiales, it is likely that this technique will be widely applicable for studying host-bacteria transcriptomics or host-microbiome metatranscriptomics.

## Methods

### *Wolbachia* and *D. ananassae* RNA Isolation

To examine the contribution of different RNAs in pre- and post-enrichment samples, we used wild-type *D. ananassae* from Klang, Selangor, Malaysia (UCSD Stock No. 14024–0371.33). Insects were reared on Jazz-Mix Drosophila food (Applied Scientific, Waltham, MA, USA) in plastic bottles in an insect growth chamber (Caron, Marietta, OH, USA) at 25 °C and 68% humidity. Natural infection by *Wolbachia* endosymbiont *w*Ana was confirmed with *Wolbachia-*specific fluorescence *in situ* hybridization on ovaries^23^ prior to total RNA extraction from ~50 adults using an RNeasy Mini Kit (Qiagen, Valencia, CA, USA), for each of three biological replicates. RNA was DNase-treated with the optional on column DNase digestion per the manufacturer’s protocol. To further remove contaminating DNA, ≤87.5 μL of the RNA sampled was combined with 10 μL Buffer RDD, 2.5 μL RNase-free DNase I (Qiagen, Valencia, CA, USA), and brought up to 100 μL with Rnase-free water. The mixture was incubated at room temperature for 10 min and then RNA purified with the Qiagen RNeasy Mini protocol following the manufacturer’s protocol.

### *Ehrlichia chaffeensis* Wakulla and canine RNA isolation

The *Ehrlichia chaffeensis* Wakulla strain (originally provided by the Centers for Disease Control and Prevention, Atlanta, GA) was propagated in a canine macrophage cell line DH82 as described previously^24^ in DMEM supplemented with 10% FBS and 2% L-glutamine. At 3 d post infection, when ~95% infected of cells were infected, the DH82 cells (~5 × 10^6^ cells) were harvested, and total RNA was extracted using an RNeasy Mini Kit (Qiagen, Valencia, CA, USA) with the optional on column DNase-digestion per the manufacturer’s protocol.

### Bacterial mRNA Enrichment

Using 5 μg total RNA, the Ribo-Zero removal protocol (Epicentre, Madison, WI, USA), was carried out with the standard amount of magnetic beads (225 μL per reaction), followed by the Invitrogen Dynabeads polyA enrichment protocol (Life Technologies, Grand Island, NY, USA) keeping the polyA depleted material in the supernatant and discarding the polyA-enriched material. The similarity in composition, pH, and ionic strength of Epicentre’s and Invitrogen’s reaction buffers allowed us to follow the protocols consecutively. Since the total RNA mixture was assumed to have a higher ratio of host rRNA compared to endosymbiont rRNA, we added 8.5 μL Human/Mouse/Rat Removal Solution to 1.5 μL Gram Negative Bacteria Removal Solution at the Ribo-Zero rRNA removal step for the standard removal. For the low input removal, we added 1.7 μL Human/Mouse/Rat Removal Solution to 0.3 μL Gram Negative Bacteria Removal Solution. The Ribo-Zero Magnetic Kit procedure was followed and, after removal of the magnetic beads, the eluate was processed with the standard Dynabeads protocol. Samples taken before and after bacterial mRNA enrichment were analyzed on an Agilent Bioanalyzer.

Using both the Agilent Bioanlayzer and qRT-PCR (below), the template added reflects the same amount of starting material. For example, if the input volume of starting material was 50 μL and the final output supernatant was 100 μL, 1 μL of the starting material was used per qRT-PCR reaction or Bioanalyzer well for the total/starting RNA sample while 2 μL of the supernatant was used for the bacterial mRNA enriched sample. This was done to reflect the dilution between the starting material and the final supernatant volumes. As a consequence, these areas of integration and the Ct values described below can be compared directly in order to evaluate the composition of the samples.

### Bacterial mRNA Enrichment Using the Low Input Method

Using 100 ng total RNA and a protocol distributed by Clontech (http://www.clontech.com/JP/Products/cDNA_Synthesis_and_Library_Construction/Next_Gen_Sequencing_Kits/ibcGetAttachment.jsp?cItemId=75952&fileId=6660677&sitex=10025:22372:US), the Ribo-Zero rRNA removal protocol (Epicentre, Madison, WI, USA) was carried out using a smaller amount of rRNA removal beads (90 μL), followed by the Invitrogen Dynabeads polyA enrichment protocol (Life Technologies, Grand Island, NY, USA) keeping the polyA-depleted material in the supernatant and discarding the poly-enriched material. Like the higher input method, the similarity of Epicentre’s and Invitrogen’s reaction buffers allowed us to follow the protocols consecutively. Since the total RNA mixture was assumed to have a higher ratio of host rRNA compared to endosymbiont rRNA, we added 1.7 μL Human/Mouse/Rat Removal Solution to 0.3 μL Gram Negative Bacteria Removal Solution at the Ribo-Zero rRNA removal step, maintaining the same removal solution ratio as the 5 μg method. The modified Ribo-Zero Magnetic Kit procedure was followed and, after removal of the magnetic beads, the eluate was processed with the standard Dynabeads protocol.

### Assessment of *Wolbachia* mRNA Enrichment by Quantitative Real-Time PCR

We examined the effectiveness of the bacterial mRNA enrichment, using a Ribo-Zero and Dynabeads based technique, by targeting host and *Wolbachia* RNA molecules whose abundance was assumed to either be decreased or unchanged by components in our protocol. The qRT-PCR primers (Table 3) were designed using Primer3 and synthesized by Sigma-Aldrich. Equivalent amounts of pre- and post-enriched RNA were used as templates such that it reflected the same amount of starting material as described above. A one-step qRT-PCR reaction containing 2× Quantitect SYBR Green, RNase-free water, and QuantiTect Reverse Transcriptase, was carried out following the manufacturer’s protocol (Qiagen, Germantown, MD, USA). The assays were conducted on an ABI 7900HT instrument (Life Technologies, Grand Island, NY, USA). The reactions were incubated at 50 °C for 30 min and then denatured at 95 °C for 15 min, followed by amplification with 45 cycles of 94 °C for 15 s, 55 °C for 30 s, and 72 °C for 30 s. Data was analyzed using a comparative cycle threshold (ΔCt) method, comparing the pre-enrichment Ct to the post-enrichment Ct for each locus tested. Each sample was tested in triplicate.

### Transcriptome Sequencing

Illumina RNA-Seq libraries were prepared with the TruSeq RNA Sample Prep kit (Illumina, San Diego, CA) per the manufacturer’s protocol. For the bacterial RNA-enriched sample, the polyA isolation step was omitted. The total RNA sample was prepared from all RNA present in the sample, while the polyA-selected library had the polyA isolation step performed. Adapters containing seven nucleotide indexes were ligated to the double-stranded cDNA. The DNA was purified between enzymatic reactions and the size selection of the library was performed with AMPure XT beads (Beckman Coulter Genomics, Danvers, MA). The libraries were pooled and sequenced on an Illumina MiSeq or Illumina HiSeq sequencer paired end run, as specified in the text.

### Sequence Read Mapping of *D. ananassae* Data

Forward and reverse reads were aligned with the BWA MEM command implemented in BWA v0.7.12 using the –M option^17^ against a database containing the *D. ananassae* and *w*Ri genomes. The alignments were sorted and duplicates removed using Picard v.1.129. Coverage across the genome was measured using SAMTOOLS MPILEUP implemented in v.0.1.19 with the options “–BQ0 –d10000000”^25^.

### Sequence Read Mapping of *E. chaffeensis* Data

Forward and reverse reads were aligned with Bowtie2^26^ using the reference Wakulla genome (CP007479.1). Alignment statistics were assessed using SAMTOOLS IDXSTAT command implemented in v.0.1.19 and coverage across the genome was measured using SAMTOOLS MPILEUP implemented in v.0.1.19 with the options” –BQ0 –d10000000”^25^.

### Data availability

The data set(s) supporting the results of this article are available in the Sequence Read Archive (SRA) repository. The *D. ananassae* datasets are available in SRP061993 http://www.ncbi.nlm.nih.gov/sra/?term=SRP061993) and the *E. chaffeensis* data set as SRX487088 (http://www.ncbi.nlm.nih.gov/sra/?term=SRX487088).

## Additional Information

**How to cite this article**: Kumar, N. *et al*. Efficient Enrichment of Bacterial mRNA from Host-Bacteria Total RNA Samples. *Sci. Rep.* **6**, 34850; doi: 10.1038/srep34850 (2016).

## Figures and Tables

**Table 1:** Transcripts assessed by qRT-PCR.

RNA Type	Removal Component	Post-enrichment Abundance	Target	Gene Description	ΔCt* Drosophila-Wolbachia Replicate 1	ΔCt Drosophila-Wolbachia Replicate 2	ΔCt Drosophila-Wolbachia Replicate 3	ΔCt Brugia-Wolbachia Replicate 1	ΔCt Brugia-Wolbachia Replicate 2
*Drosophila* mRNA	Dynabeads	Decreased	Act5c	Actin 5C	−1.98	−1.30	−2.12	NA***	NA
*Drosophila* mRNA	Dynabeads	Decreased	RpL32 exon	Rbosomal protein L32 (RPL32) within an exon	−3.06	−2.41	−3.38	NA	NA
*Drosophila* mRNA	Dynabeads	Decreased	RpL32 boundary	Ribosomal protein L32 (RPL32) across intron/exon boundary	−3.08	−2.51	−3.37	NA	NA
*Drosophila* rRNA	Ribo-Zero (H/M/R)	Decreased	28S rRNA	*Drosophila ananassae* 28S ribosomal RNA	NQ**	NQ	NQ	NA	NA
*Drosophila* rRNA	Ribo-Zero (H/M/R)	Decreased	18S rRNA	*Drosophila ananassae* 18S ribosomal RNA	NQ	NQ	NQ	NA	NA
*Wolbachia w*Ana rRNA	Ribo-Zero (Gram-Neg)	Decreased	16S rRNA	*Wolbachia* strain *w*Ri 16S ribosomal RNA gene	−5.48	−13.9	−13.9	−7.06	−8.00
*Wolbachia w*Ana mRNA	No removal	No change	WD_1289	Ribosomal protein S10	0.17	0.475	0.69	NA	NA
*Wolbachia w*Ana mRNA	No removal	No change	WD_0443	Hypothetical protein	0.66	−0.33	−0.02	NA	NA
*Wolbachia w*Ana mRNA	No removal	No change	WD_0880	Coenzyme PQQ synthesis protein C	0.69	−0.15	0.36	NA	NA
*Brugia* mRNA	Dynabeads	Decreased	Actin	actin (partial mRNA)	NA	NA	NA	−2.08	−2.68
*Brugia* mRNA	Dynabeads	Decreased	Bm1_03910	40S ribosomal protein S27	NA	NA	NA	−3.02	−3.95
*Brugia* rRNA	Ribo-Zero (H/M/R)	Decreased	Bm_18S	*Brugia malayi* 18S ribosomal RNA	NA	NA	NA	−16.4	−17.4
*Wolbachia w*Bm mRNA	No removal	No change	Wbm0276	DnaA from *Wolbachia* strain *w*Bm	NA	NA	NA	0.91	−1.02
*Wolbachia w*Bm mRNA	No removal	No change	Wbm0350	GRoEL from *Wolbachia* strain *w*Bm	NA	NA	NA	0.84	−1.16

^*^The ΔCt was calculated as the difference between the Ct value of the original RNA sample and the sample following bacterial mRNA enrichment such that a negative value reflects loss of the molecule being tested.

^**^NQ = not quantifiable. More specifically, values for the *Drosophila* rRNA are not shown because they are not in the linear range under these conditions. Modifying the conditions to make them linear would have prevented the detection of the *Wolbachia* mRNA. However, a large not quantifiable decrease in the Ct was also observed for the *Drosophila* rRNA following rRNA depletion.

^***^NA = not applicable.

**Table 2:** Approximate Costs.

	Standard Input Bacterial mRNA Enrichment	Low Input Bacterial mRNA Enrichment	Standard rRNA Reduction	Low Input rRNA Reduction	No reduction	Agilent SureSelect
Ribo-Zero Human/Mouse/Rat*	$64.81	$16.01	$64.81	$16.01	$0	$0
Ribo-Zero Gram Negative Bacteria**	$12.88	$3.22	$12.88	$3.22	$0	$0
DynaBeads***	$46.20	$46.20	$0	$0	$0	$0
Agilent SureSelect RNA capture****	$0	$0	$0	$0	$0	$562.50
mRNA library construction	$325	$325	$325	$325	$325	$325
Sequencing costs (insect-bacteria)*****	$12,000	$12,000	$12,000	$12,000	$600,000	—
Total (insect-bacteria)	$12,448.89	$12,390.43	$12,402.69	$12,344.23	—	—
Sequencing costs (canine-bacteria)******	$839	$839	$839	$839	—	—
Total (canine-bacteria)	$1,288.05	$1,229.59	$1,241.85	$1,183.39	—	—

^*^Based on a $1,830 kit of 24 reactions and using 8.5 μL for the standard input and 1.7 μL for the low input, as described in the methods.

^**^Based on a $515 kit of 6 reactions and using 1.5 μL in the standard input and 0.3 μL in the low input, as described in the methods.

^***^Based on a $462 kit of 10 reactions.

^****^Based on the $7,500 list price for 16 reactions of a Tier 2 custom capture of a 0.5–2.9 Mbp genome and a $1,500 reagent kit.

^*****^Based on a $2,400 100-bp paired end HiSeq channel generating 200 million read pairs and targeting the acquisition of 10 million mapped bacterial read pairs/transcriptome, using the *Drosophila*-*Wolbachia* sequencing results, specifically that 1.0% of reads matched *Wolbachia* sequences as opposed to 0.02% in the total RNA; In reality the samples without reduction would perform markedly worse since the reads would most likely map to the rRNA.

^******^Based on a $2,400 100-bp paired end HiSeq channel generating 200 million read pairs and targeting the acquisition of 10 million mapped bacterial read pairs/transcriptome, using the *Ehrlichia*-canine sequencing results, specifically that 14.3% of reads mapped to *Ehrlichia* following enrichment.

**Table 3:** Primer sequences.

Name	Forward Name	Forward Sequence	Reverse Name	Reverse Sequence	Size
Act5c	Act5CF	GTCATCTTCTCACGGTTAGC	Act5CR	AGATCTGGCATCACACCTTC	109
RpL32 exon	RpL32_exonF	TCGCTTCAAGGGTCAGTACC	RpL32_exonR	TCTGCATCAGCAGGACCTC	134
RpL32 boundary	RpL32_boundaryF	CGAAGTTGTCGCACAAATGG	RpL32_boundaryR	GGTGCGCTTGTTGGAACC	113
Dana_UNIQ_1	Dana_UNIQ_1_F	CTGAGCTGCGAATACTGCAC	Dana_UNIQ_1_R	CAAGTCCGGCTTAATCTTGG	186
Dana_28S	Dana_28S_F	CCAAAGAGTCGTGTTGCTTG	Dana_28S_R	AACGGATATTCAGGTTCATCG	187
Dana_18S	Dana_18S_F	TGGTCTTGTACCGACGACAG	Dana_18S_R	GCTGCCTTCCTTAGATGTGG	156
Wana_16S	Wana_16S_F	GCTCGTGTCGTGAGATGTTG	Wana_16S_R	AAGGGCCATGATGACTTGAC	146
WD_qPCR_1289	WD_qPCR_1289F	TTTGCACTCGGTGCATTTAC	WD_qPCR_1289R	CAAGCAGTTGCACCATTTTTAC	101
WD_qPCR_0443	WD_qPCR_0443F	TGCAATTGCCAATGGTTATG	WD_qPCR_0443R	ATTCTGCCTTCAACGTCAGG	117
WD_qPCR_0880	WD_qPCR_0880F	AATGGCATTCTGAGGAATGTG	WD_qPCR_0880R	CAAGAAAACCCCACAAGAGC	111
WD_qPCR_1012	WD_qPCR_1012F	AGCGAAAGATGGAAGTGGTG	WD_qPCR_1012R	CATTTCCTTCCACTCCAAGC	139
WD_qPCR_1094	WD_qPCR_1094F	GGAAACGAGGAATTAATCAAGC	WD_qPCR_1094R	CCTGTTCCATCGCAGTAACC	104
Actin	Actin_F	TGCTGATCGTATGCAGAAGG	Actin_R	GGAGAGTGACGCCAGGATAG	124
Bm1_03910	Bm1_03910_F	GCCGTTAGCACGAGATTTATTG	Bm1_03910_R	AGGGCACTTTACATCCATGAAG	112
Wbm0276	Wbm0276_F	GGTTCGCCACTAGATCCAAG	Wbm0276_R	CCCACTCCGCCATATAGAAAC	146
Wbm0350	Wbm0350_F	CGTTGCTGTGCTTAAAGTCG	Wbm0350_R	AAGTGCAACTCCACCACCTG	133
Bm_18S	Bm_18S_F	ACTTCATGCGGCTAAACACC	Bm_18S_R	TGGTGGAGTGATTTGTCTGG	124
